# Modeling Tool for Decision Support during Early Days of an Anthrax Event 

**DOI:** 10.3201/eid2301.151787

**Published:** 2017-01

**Authors:** Gabriel Rainisch, Martin I. Meltzer, Sean Shadomy, William A. Bower, Nathaniel Hupert

**Affiliations:** Centers for Disease Control and Prevention, Atlanta, Georgia, USA (G. Rainisch, M.I. Meltzer, S. Shadomy, W.A. Bower, N. Hupert);; Weill Cornell Medical College and New York–Presbyterian Hospital, New York, New York, USA (N. Hupert)

**Keywords:** Keywords: Bacillus anthracis, anthrax, inhalation anthrax, bioterrorism and preparedness, biohazard release, Sverdlovsk accidental release, biological warfare, mass casualty incidents, theoretical models, decision support models, public health practice, chemoprophylaxis, bacteria

## Abstract

Health officials lack field-implementable tools for forecasting the effects that a large-scale release of *Bacillus anthracis* spores would have on public health and hospitals. We created a modeling tool (combining inhalational anthrax caseload projections based on initial case reports, effects of variable postexposure prophylaxis campaigns, and healthcare facility surge capacity requirements) to project hospitalizations and casualties from a newly detected inhalation anthrax event, and we examined the consequences of intervention choices. With only 3 days of case counts, the model can predict final attack sizes for simulated Sverdlovsk-like events (1979 USSR) with sufficient accuracy for decision making and confirms the value of early postexposure prophylaxis initiation. According to a baseline scenario, hospital treatment volume peaks 15 days after exposure, deaths peak earlier (day 5), and recovery peaks later (day 23). This tool gives public health, hospital, and emergency planners scenario-specific information for developing quantitative response plans for this threat.

Population exposure to aerosolized *Bacillus anthracis* spores is one of the most potentially catastrophic public health emergencies (*1*). The 2001 US anthrax attack, in which inhalation anthrax (IA) affected 11 persons and killed 5, led to multiple mass antimicrobial prophylaxis campaigns and considerable healthcare activity (*2*). Data in the first few days of such an event may be limited, leading to uncertainty regarding the scale of the event and difficulty making response decisions.

Public health officials lack widely available tools for rapidly estimating the number of cases, projecting medical surge, and evaluating response options during an anthrax event. Several efforts have evaluated response options in predefined scenarios, which are useful for planning but not during a response (*3*–*6*). Two other models have attempted to predict the number and timing of IA cases after exposure to aerosolized *B. anthracis* spores; 1 evaluated response options (*7*,*8*). However, neither model estimates the surge of patients in the healthcare system, and both models have constraints that limit their practical utility. Walden and Kaplan built a model that presumes equal probability of various event sizes and requires at least 5 days of case data before robust estimates of final attack sizes can be calculated (*8*). This timing may be insufficient given the US Cities Readiness Initiative (CRI) guideline that postexposure prophylaxis (PEP) dispensing be completed within 48 hours of event detection (*9*). The back-calculation techniques of Egan et al. permit estimation of the final outbreak size after a certain number of observed cases under different PEP assumptions (*7*,*10*). Although these models can be reconciled with the CRI timeline, they were not designed for direct use by public health practitioners (use requires the R coding language and understanding of maximum-likelihood functions), and the earlier work assumes 90% PEP uptake by the infected population, which is an overestimation (>25%) of the probable public response (*11*). 

An alternative method for predicting the scale of IA events is plume modeling, which calculates the number of exposed persons by estimating the geographic spread of dispersed *B. anthracis* spores. Plume models require knowledge (or estimates) of the number of spores released, release timing and location, population densities, meteorologic data (e.g. wind speed and direction), and inhaled spore volume. It is unclear whether plume modeling is sufficiently timely and robust to guide local response decisions.

We therefore developed a modeling tool, called Anthrax Assist, to provide public health officials with rapid projections of IA cases and response decision support during an aerosolized anthrax event. This tool can assist with responding to an anthrax event (or designing and conducting locally tailored training exercises) by providing critical information in the first few days of response.

## Methods

### Tool Overview

We used Excel 2010 (Microsoft Corporation, Redmond, WA, USA) to construct Anthrax Assist (Technical Appendix 1). Anthrax Assist is composed of 3 linked models (Table 1). The Epidemic-Curve model combines daily case counts with incubation distributions to project the future number and timing of symptomatic IA cases in a nonvaccinated population. The PEP Impact model estimates the potential decrease in the projected trajectory of future cases (output from the Epidemic-Curve model) resulting from a PEP dispensing campaign. The Healthcare Impact model uses the projected unmitigated or PEP-mitigated incidence curves to project the size and timing of peak healthcare utilization and associated patient outcomes. Users can readily change a number of input values to reflect a desired attack scenario or response strategy (Table 2). To illustrate the models, we developed an attack scenario and used it to evaluate estimates resulting from various outbreak detection scenarios (using 1, 2, or 3 days of initial case count data) and PEP response strategies (Table 3).

**Table 1:** Anthrax Assist models and associated inputs, outputs, and public health decisions supported*

Model	Inputs	Outputs	Decision informed
Epidemic Curve	1) Case counts by illness-onset date	1) Cumulative caseload	How the event unfolds:
	2) Incubation period distribution	2) Unmitigated epidemic curve	1) Size of event
			2) How quickly people become ill
PEP Impact	1) Epidemic curve (output from Epidemic Curve model)	1) Cases prevented by PEP	1) Initiate a PEP campaign and when to begin
	2) Dispensing plan	2) PEP-mitigated epidemic curve	2) How much PEP to dispense
	3) Effectiveness		3) Dispensing resource requirements
	4) Population needing prophylaxis		
Healthcare Impact	1) Unmitigated epidemic curve (output from Epidemic Curve model) or PEP-mitigated epidemic curve (output from PEP Impact model)	1) Hospital demand curves:	1) Treatment guidance:
		a) ED surge	a) messaging to public
		b) treatment load	b) standards of care
	2) Disease progression	2) Deaths curve	2) Set treatment priorities
	3) Treatment-seeking behavior	3) Recovered curve	3) Mobilize medical care resources
	4) Treatment effectiveness and availability		

*ED, emergency department; PEP, postexposure prophylaxis.

**Table 2:** Inputs and parameter values for all Anthrax Assist models*

Parameter	Baseline value	Range†	User adjustable‡	Reference
Epidemic-Curve model				
Case counts for days 1, 2, 3§	20, 10, 70	1–4 days of data	Yes	(*12*)
Median inhaled spore count, no.¶	360	1–8,000	Yes	(*13**,**14*)
Median incubation, d ± SD	6.9 ± 1.8	10.3–5.0 ± 2.2–1.6	Yes	(*13*)
Population size of the impacted jurisdiction, no.	500,000		Yes	Assumed
PEP Impact model				
Size of population to receive prophylaxis	500,000		Yes#	Assumed
PEP throughput at full capacity, daily	250,000		Yes	Assumed**
Delay to PEP campaign start, d††	2	1–2	Yes	(*9*)
Ramp-up period until PEP campaign throughput reaches full capacity, d	0		Yes	Assumed**
PEP campaign duration at full throughput capacity, d	2	1–4	Yes	Assumed**
PEP uptake, %‡‡	65	40–90	Yes	(*11*)
Antibiotic efficacy, %	90		Yes	(*15**–**17*)
Adherence to PEP regimen at event day 60, %	40	25–80	Yes	(*18*)
Time until antimicrobials are protective, d	1		No	(*15**–**17*)
Healthcare Impact model				
Public health messaging starts, d of event§§	2		Yes	Assumed
Proportion seeking care relative to public health message timing, by disease state				(*2*)
During prodromal stage, %	40 before; 80 after		Yes	
During fulminant stage, %	95 before; 95 after		Yes	
Daily transition fraction from prodromal to fulminant illness, by outcome				(*19*)
Eventually recover, %	20		No	
Eventually die, %	50		No	
Maximal length of prodromal illness, by outcome				(*19*)
Eventually recover, d	5		No	
Eventually die, d	2		No	
Length of fulminant illness among untreated, d	0		No	Assumed
Length of fulminant illness among treated who die, d¶¶	1		No	(*19*)
Median ± SD of normal distribution of length of treatment among those who recover, d¶¶	18 ± 3		No	(*19*)
Recover with treatment, by stage of illness when treatment initiated, %##				Assumed
Prodromal, %	80		Yes	
Fulminant, %	20		Yes	
Prodromal who recover after fulminant illness, %***	50		Yes	(*2*)

*Amerithrax, anthrax attacks in the United States during 2001; CRI, Cities Readiness Initiative; PEP, postexposure prophylaxis. †Values provided were used in our evaluation of the influence of the number of days of case data on Epi-Curve projections (case counts parameter), to create high and low final case count estimates (median inhaled spore count and median incubation parameters), and to evaluate various PEP scenarios (all PEP-Impact model parameters) (Table 3). Range values used in the univariate sensitivity analysis of PEP parameters differ (Table 4). ‡Anthrax Assist user can readily change the input value. §Case counts from the first 3 days of the 1979 Sverdlovsk (USSR) anthrax event epidemic curve inflated by a factor of 10. When 4 days of case counts are used (Table 4), the fourth day of counts is 40. ¶360 spores is a dosage estimated to have occurred during the 1979 Sverdlovsk (USSR) anthrax event (*13*). One spore represents the minimum possible infectious dose, and 8,000 is a plausible high dose (*14*). #Cannot exceed the value of the Epidemic-Curve model “Population size of the impacted jurisdiction” parameter. When less, proportionately fewer infected persons are eligible for PEP protection. **Value chosen so that PEP dispensing is in accordance with US CRI guidelines and is completed within 2 days after the decision to initiate PEP (9). ††Determined by counting days from date of earliest illness onset (i.e., event day 1). ‡‡Percentage of population targeted to receive PEP who actually obtain and start PEP. §§Public health messaging only influences treatment-seeking behavior in the absence of a PEP campaign or prior to campaign initiation. ¶¶Same length assumed for patients initiating treatment in the fulminant versus prodromal stage of illness. ##Assumes an improved treatment effectiveness compared with the 2001 US anthrax attacks as a result of clinical experience gained in treating inhalation anthrax cases in the United States since and the recent availability of intravenous antitoxin; in addition to the full complement of medical resources used during the 2001 attacks: an acute-care bed and the associated medical care staff (including respiratory therapists), pleural fluid drainage, mechanical respiratory ventilation, and intravenous antimicrobial drugs. In the United States in 2001, 6 (67%) of 9 persons who sought treatment during the prodromal stage of illness recovered (however, 2 who died did not receive antimicrobial drugs with activity against *Bacillus anthracis* until they exhibited fulminant illness), and both persons who sought care during fulminant illness died (*2**,**20*). ***On the basis of 6 survivors during the 2001 Amerithrax attacks who sought treatment during the prodromal illness stage: cases 2, 3, 4, 7, 8, 9 (*2**,**20*). Progression to fulminant illness was defined as severe symptomatic disease characterized by respiratory distress requiring pleural effusion drainage, or mechanical ventilation, marked cyanosis, shock, or meningoencephalitis.

**Table 3:** PEP scenarios, by campaign logistics and antimicrobial drug use components*

Scenario (description)	Logistics components	Drug-use components
Scenario 1 (no PEP)	Not applicable	Not applicable
Scenario 2 (ideal)	1-day delay,† 1-day campaign	90% uptake,‡ 80% adherence§
Scenario 3 (practical: logistics follow CRI guidance, and utilization data based on the Amerithrax attacks)	2-day delay,† 2-day campaign	65% uptake,‡ 40% adherence§
Scenario 4 (constrained)	2-day delay,† 4-day campaign	40% uptake,‡ 25% adherence§

*Amerithrax, anthrax attacks in the United States during 2001; CRI, Cities Readiness Initiative; PEP, postexposure prophylaxis. †Delay days are determined by counting the days from the date of earliest illness onset (i.e., event day 1). Public health messaging also begins on the same day as the campaign. The delay dictates the number of days of case data potentially available as input. Two days of case data are available as input in Scenario 2, and 3 days are available as input in Scenarios 3 and 4.  ‡Proportion of the population targeted by public health officials to receive PEP who actually obtain and start PEP (*11*). §Proportion fully adhering to the PEP regimen on event day 60 (*18*).

### Calculations

#### Epidemic-Curve Model

We base our IA incubation distribution on the Wilkening model, which plots the probability of becoming symptomatic over a 60-day period for a given infectious dose of *B. anthracis* spores (Technical Appendix 2) (*13*). We combine this incubation probability distribution with the number of detected IA cases at a given time to calculate the total projected number of ill persons (final case count [FCC]) by using the following equation: 

FCC = no. cases detected by day *t* /
proportion of infected persons expected to become symptomatic by day
*t, *where *t* is the number of days from
the date of the first symptomatic case to the time of analysis. The
numerator is obtained through public health disease surveillance, and the
denominator is obtained from the incubation probability distribution. We
then generate an epidemic curve by distributing the FCC over each day of the
outbreak according to the incubation probability
distribution.

We assume a single, localized release that causes near-simultaneous population exposure. Because public health authorities will probably not know the average inhaled spore dose among affected persons, we designed the model to calculate a range of plausible outbreak sizes from a range of spores inhaled per person. To illustrate the model, we used a median value of 360 spores/person (range 1–8,000), resulting in a median incubation period of 6.9 days (range 10.3–5.0) (Table 2; Technical Appendix 2).

#### PEP Impact Model

The PEP Impact model uses median projected daily case counts (output from the Epidemic Curve model) to estimate the potential effects of a PEP campaign. This effect is calculated as the product of the number of persons who become symptomatic on any given day *t*; the effectiveness of PEP on day *t* (which is a product of antimicrobial efficacy and adherence); and the probability that an infected, asymptomatic person receives antimicrobial prophylaxis on or before day *t*. We calculate the probability that a person receives PEP on day *t* by multiplying the PEP uptake (proportion of persons seeking antimicrobial drugs) by the daily antimicrobial dispensing throughput and then dividing by the population targeted for PEP (Table 2). The FCC with a PEP campaign is the sum of detected cases and daily PEP-mitigated case count projections. We express PEP effect as both a difference measure (cases averted) and as a proportion (cases averted divided by the unmitigated FCC). We assume that symptomatic persons seeking PEP are referred for medical treatment and do not receive PEP (*2**1*). We further assume that all of the population suspected to be exposed would be targeted for PEP because there is no definitive PEP triage process for IA beyond exposure risk (Table 2).

In accordance with US CRI guidelines, we assume that PEP dispensing is completed in 2 (range 1–2) days after the decision to initiate PEP (*9*). Following SteelFisher et al., we also assume that of the population targeted to receive PEP, 65% (range 40%–90%) actually start taking PEP (*11*). Everyone starting PEP is assumed to fully adhere to the regimen on the first day. After that, adherence decreases linearly to 40% (range 25%–80%) at the conclusion of the event (Technical Appendix 2) (*18*). Last, we assumed 90% (range 10%–90%) antimicrobial drug efficacy and that this level of protection is achieved 1 day after initiation of the regimen (*15*,*16*) (Table 2).

#### Healthcare Impact Model

**Figure 1:**
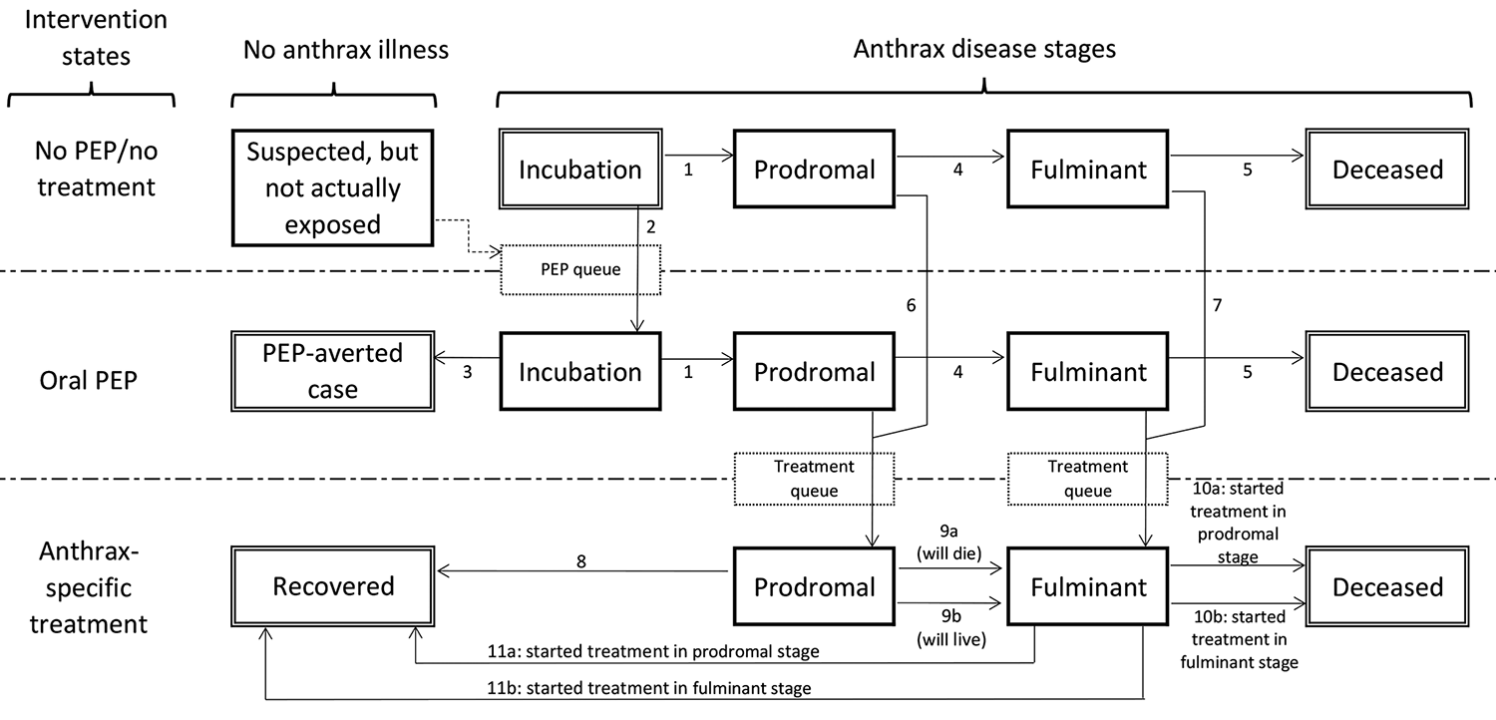
Anthrax Assist model disease stages, intervention states, and transitions. Persons begin in the top Incubation state and may transition via the numbered arrows from one state to another until they eventually reach an outcome state (doubled-walled boxes). All persons with untreated infection will progress to deceased. Recovery is possible only through effective oral PEP (averted case) or anthrax-specific treatment (recovered). Transitions are governed by the 3 Anthrax Assist models as follows: Epidemic-Curve model, transition 1; PEP Impact model, transitions 2 and 3; Healthcare Impact model, transitions 4–11. Suspected, but Not Actually Exposed cases are shown here because of their role in diluting the incubating population seeking PEP (dashed transition arrow). PEP and Treatment queues (dashed outline boxes) are depicted to reflect the necessary interactions persons must have with the public health and healthcare systems to transition between treatment states. PEP, postexposure prophylaxis.

To calculate the demand for medical care, we used a compartmental model (based on one reported by Zaric et al.) and used the review of IA cases by Holty et al. to select the rates of patients’ transitions through illness stages (*6*,*19*) (Figure 1; Technical Appendix 2). This model is used to calculate daily patients initiating treatment, peak daily treatment caseload (i.e., census of hospitalized patients receiving treatment for IA), and the day of peak treatment caseload.

In this model, medical intervention is required for recovery from symptomatic IA, and only patients with fulminant disease can die. We define treatment effectiveness as the percentage of patients who recover after receiving some type of medical intervention and pattern it after the 2001 US IA events. As such, treatment is 4 times more effective when started in the prodromal (80%), rather than fulminant (20%), stage of illness (Table 2). However, the probability that a patient in the fulminant stage seeks healthcare (95%) is roughly twice that for someone in the prodromal stage (40%) (*22*). In addition, we varied the likelihood that any patient seeks healthcare by the timing of public health messaging regarding screening and treatment recommendations. We assume that the proportion of persons in the prodromal stage who seek care would double as a result of widespread media attention (80% vs. 40%) (*2*) (Table 2). Last, we assume treatment effectiveness values based on full availability of medical countermeasures and resources at the time of treatment and no delay in access to care once sought (Table 2).

During the 2001 US IA event, treatment duration was highly associated with treatment outcomes (*22*). Thus, for those who recover, we assume a normal distribution with a mean of 18 (SD 3) treatment days from the date of transition to the fulminant stage of illness or from the sixth day of prodromal illness for patients whose illness does not progress to the fulminant stage. For patients who eventually recover from fulminant illness (in treated and yet-nontreated populations), we assume a 20% transition each day so that all have transitioned to the fulminant stage after 5 days in the prodromal stage. Among those who eventually die, half transition to the fulminant stage on the first day of symptoms and the other half on the next day. When treatment is not sought, we assume that death occurs on the same day as the transition to fulminant illness.

### Scenarios

To illustrate use of the models, we created an attack case series scenario patterned after the 1979 Sverdlovsk, USSR, event, in which at least 70 people died of IA after accidental aerosol release of *B. anthracis* spores from a bioweapons facility (Table 2) (*12*). We created this Sverdlovsk-like case series by multiplying each day’s case count from the Sverdlovsk event by 10, resulting in a 40-day, 700-patient case series (Technical Appendix 2).

To illustrate the accuracy of the Anthrax Assist FCC projections under realistic conditions of limited reported case data in the first days of an event, we first ran the Epidemic-Curve portion of Anthrax Assist by using only the first 3 days of case data as input (20, 10, and 70 cases, respectively), then by using 2 days of case data, and then only the first day’s cases. To examine the effect of the number of days of case data on the accuracy of our FCC projection, we also incrementally added a day of case data, beyond the first 3 days, until the projection was within 10% of the true FCC.

Next, to evaluate prophylaxis response options, we developed 4 PEP scenarios by varying components of the PEP campaign implementation (logistics) and the public response to the campaign (utilization) (Table 3). Scenario 1 (no PEP) is an event without a PEP campaign. Scenario 2 (ideal) is an event wherein early detection of the event (e.g., through biosensors) and positive public perception results in a 1-day campaign starting 1 day after detection, 90% uptake, and 80% adherence at the event’s conclusion. Scenario 3 (practical) is an event in which PEP dispensing logistics follow current public health guidance and PEP utilization is based on data from the 2001 US IA event, resulting in a 2-day campaign starting 2 days after detection, 65% uptake, and 40% adherence at the event conclusion. Scenario 4 (constrained) is an event in which logistics hurdles (e.g. staffing shortages, traffic congestion [*3*,*23*]) and poor public perception impede rapid PEP coverage, resulting in a 4-day campaign starting 2 days after detection, 40% uptake, and 25% adherence at event conclusion. Hereafter, the baseline scenario comprises PEP scenario 3 and the Healthcare Impact model values in Table 2.

### Sensitivity Analyses

We conducted 2 sensitivity analyses. We first evaluated the influence of individual PEP-related parameters on outputs from the models as follows: prophylaxis campaign duration of 1–6 days at full throughput capacity, delay of 3–6 days until PEP campaign starts, a range of 15%–90% for PEP uptake, a range of 10%–90% for antimicrobial efficacy, and a range of 15%–90% for adherence to the regimen at the conclusion of the event. These ranges encompass reported values (*3*,*4*,*11*,*18*,*24*).

In our second sensitivity analysis, we altered the Epidemic-Curve model inputs used in the baseline attack scenario to illustrate potential data limitations and surveillance inaccuracies that might occur during an actual event. Doing so involved comparing estimates using the full complement of the initial 3 days of case data with a scenario in which 60% of cases are reported. This level of underreporting represents the plausible difficulties often encountered when initially collecting outbreak data.

## Results

**Figure 2:**
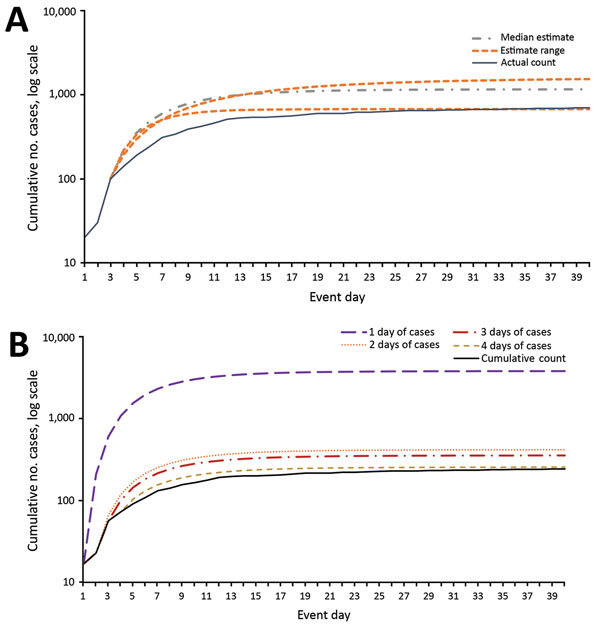
Comparison of the estimated cumulative epidemic curve by using 3 days of surveillance data with the actual event curve (A), and comparison of the median estimated cumulative epidemic curve with the actual event curve (B), by days of surveillance data available. Actual case data are case counts from the 1979 Sverdlovsk (USSR) anthrax outbreak (*12*), inflated by a factor of 10. Estimates were produced by using the first days of case data from that event (20 cases on day 1, 10 on day 2, 70 on day 3, and 40 on day 4) and other Epidemic-Curve model values listed in Table 2.

For the scenario that uses the first 3 days of case data, no PEP campaign, and early public messaging, the tool projects a median 60-day FCC of 1,164 (66% higher than actual FCC, plausible range 675–1,612; Figure 2, panel A), 35% event mortality (408 deaths), and a peak hospital caseload of 692 patients on day 15 (Table 5). Running the same scenario with only 2 days of case data (i.e., 20 followed by 10 cases) yields a median FCC estimate of 1,441 (106% higher than actual FCC, range 963–1,464) (Figure 2, panel B), 35% event mortality (506 deaths), and a peak hospital caseload of 856 on day 14. Using only the first day of case data (20 cases) yields a median FCC estimate of 27,555 (3,800% greater than actual FCC, range 10,993–36,603), 35% event mortality (1,688 deaths), and a peak hospital caseload of 2,871 on day 14. In contrast, when 4 days of case data are used, the FCC projection (median 750, range 435–1,175) falls within 10% of actual FCC.

**Table 5:** PEP effects by number of days of surveillance data available and different scenarios of PEP distribution, uptake, and adherence*

Days of baseline case data, scenario	Median projected caseload, no.	Cases averted by PEP, no. (%)	Peak treatment load, no.	Median projected deaths, no.	Deaths averted by PEP, no. (%)
2†					
Scenario 1 (no PEP)	1,441	Not applicable	856	506	Not applicable
Scenario 2 (ideal)	324	1,117 (79)	188	124	382 (75)
Scenario 3 (practical)	760	681 (48)	447	279	227 (45)
Scenario 4 (constrained)	1,084	358 (25)	648	385	121 (24)
3‡					
Scenario 1 (no PEP)	1,164	Not applicable	692	408	Not applicable
Scenario 2 (ideal)	323	841 (79)	191	123	283 (70)
Scenario 3 (practical)	614	550 (52)	363	225	183 (45)
Scenario 4 (constrained)	875	289 (27)	521	316	92 (23)
4§					
Scenario 1 (no PEP)	750	Not applicable	440	270	Not applicable
Scenario 2 (ideal)	269	481 (79)	161	103	165 (62)
Scenario 3 (practical)	481	332 (54)	244	163	107 (40)
Scenario 4 (constrained)	572	178 (29)	334	215	55 (20)

*Case data are from the 1979 Sverdlovsk (USSR) anthrax outbreak (*12*), inflated by a factor of 10. Estimates were calculated by using values shown in Table 2, except for the selected PEP Impact model parameters varied to create the PEP scenarios analyzed here: these are identified in Table 3. PEP, postexposure prophylaxis. †Day 1, 20 cases; day 2, 10 cases. ‡Day 1, 20 cases; day 2, 10 cases; day 3, 70 cases. §Day 1, 20 cases; day 2, 10 cases; day 3, 70 cases; day 4, 40 cases.

Irrespective of the number of days of case data available, the estimated effects of PEP ranged from ≈25% cases averted in scenario 4 (constrained) to 79% in scenario 2 (ideal) (54). These PEP effects are equally reflected in the percentage of averted deaths (Table 5). Even with rapid event detection, an aggressive PEP campaign, and unlimited treatment resources one-third of deaths expected under the unmitigated scenario will still occur (calculated as the ratio of deaths in PEP scenario 2 [ideal] to deaths in PEP scenario 1 [no PEP], using 3 days of case data) (Table 5).

**Figure 3:**
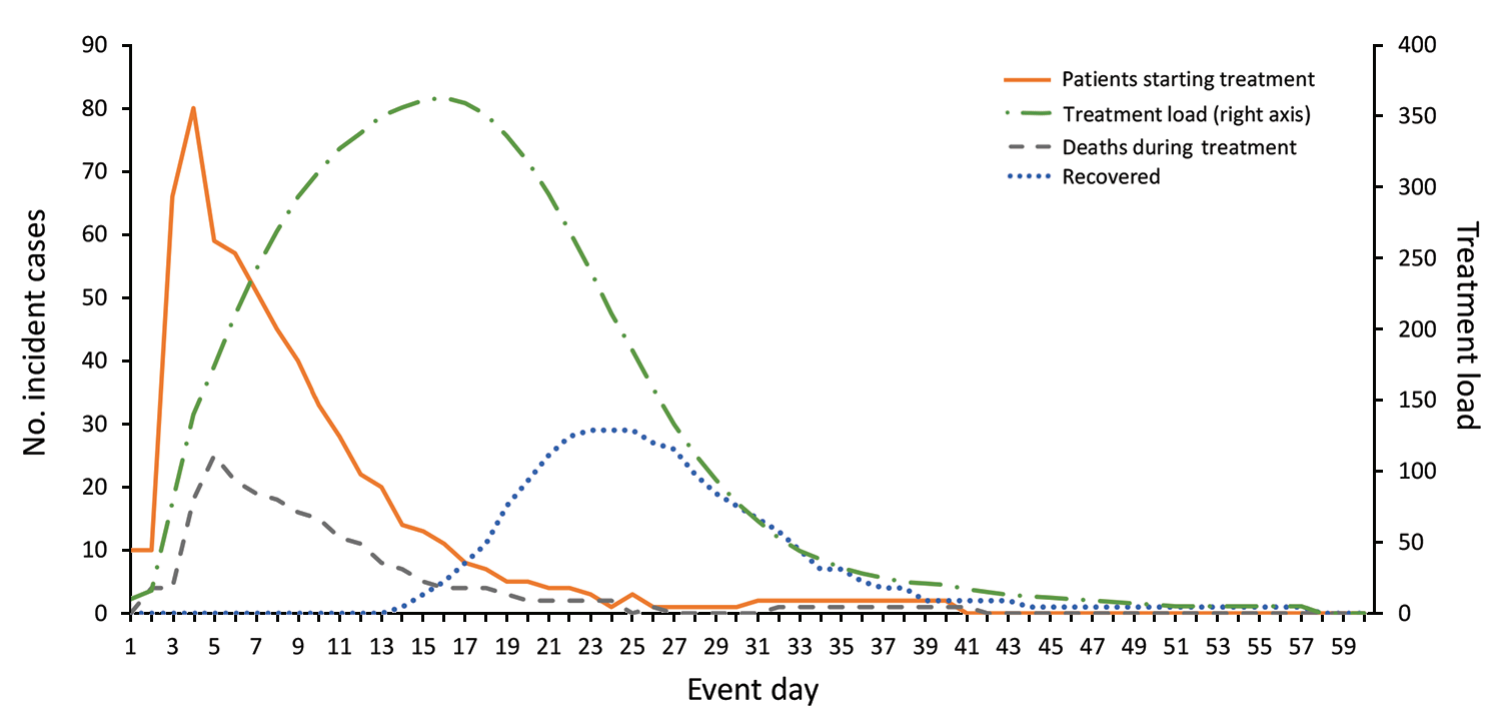
Projected daily patients seeking treatment, daily treatment load, and treatment outcomes by event day (baseline scenario). Estimates were calculated by using values shown in Table 2. Base case scenario is the same as PEP Evaluation Scenario 3 (practical) (Table 3). PEP, postexposure prophylaxis.

In the baseline scenario, treatment initiation and deaths peak early in the event (days 4 and 5, respectively), and treatment load and recoveries peak later (days 15 and 23, respectively) (Figure 3). The treatment load curve exhibits a plateau-like shape because of the extended length of time required to treat and recover from IA.

### Sensitivity Analysis

#### Influence of Individual PEP Campaign Factors

**Figure 4:**
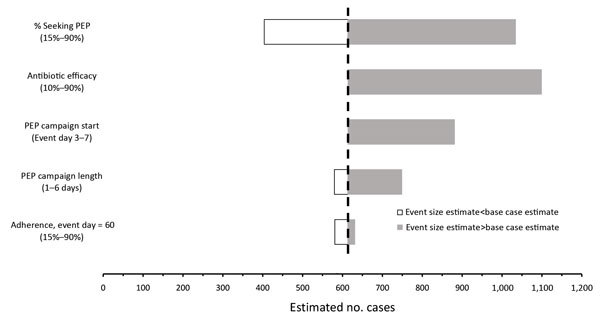
Final case count estimates comparisons to the baseline scenario estimate (614 cases) for selected PEP campaign parameter ranges. The base case estimate was produced using data from the first 3 days of the 1979 Sverdlovsk (USSR) anthrax outbreak (*12*), inflated by a factor of 10. All other values used in calculations are shown in Table 2. PEP, postexposure prophylaxis.

The decision to take PEP (uptake) is the most influential PEP-related parameter (Figure 4). Projected cases averted differ as much as 59% when results using the lowest and highest plausible PEP uptake values (12% and 71%, respectively) are compared (Table 4). In contrast, adherence (at day 60) to the PEP regimen exhibits the least direct influence on averted cases and deaths. Averted cases differ by only 5% when the lowest and highest plausible adherence values are used (50% and 55%, respectively) in the baseline scenario. This small difference results from the fact that most infected persons become symptomatic well before declining adherence can affect PEP effectiveness (Technical Appendix 2).

**Table 4:** Effects of individual PEP campaign factors*

Variable	Median projected caseload with PEP campaign, no.	Projected averted cases from campaign start, no. (%)†	Projected averted deaths, no. (%)**‡**	Peak hospitalizations, no.
Days required to provide PEP to entire target population				
1	580	583 (55)	190 (47)	339
2§	614	550 (52)	183 (45)	363
3	651	513 (48)	166 (41)	385
4	680	483 (46)	160 (39)	405
5	723	441 (41)	142 (35)	431
6	749	415 (39)	135 (33)	448
Delay to PEP campaign start, d¶				
2§	614	550 (52)	183 (45)	363
3	681	482 (45)	156 (38)	402
4	753	411 (39)	131 (32)	450
5	821	343 (32)	107 (26)	494
6	881	283 (27)	88 (22)	535
PEP uptake, %#				
15	1,034	130 (12)	47 (12)	618
40	824	340 (32)	111 (27)	489
65§	614	550 (52)	183 (45)	363
90	404	760 (71)	259 (63)	235
Antimicrobial efficacy, %				
10	1,099	64 (6)	19 (5)	653
50	857	307 (29)	97 (24)	508
90§	614	550 (52)	183 (45)	363
Adherence to regimen at event day 60, %				
15	631	533 (50)	174 (43)	370
40§	614	550 (52)	183 (45)	363
65	597	566 (53)	184 (45)	353
90	581	583 (55)	192 (47)	342

*Estimates were calculated by using values shown in Table 2. Base case scenario is the same as PEP evaluation scenario 3 (practical) using 3 days of case data (Table 3). Without a PEP campaign, the median projected caseload would be 1,164 (Table 3, Scenario 1 [no PEP]) using 3 days of case data. PEP, postexposure prophylaxis. †% = PEP averted cases/(median attack size estimate without a PEP campaign – cases detected to date) ‡% = PEP averted deaths/(median attack size deaths estimate without a PEP campaign). This calculation assumes no deaths within the first 3 event days. §Baseline scenario value** (**Table 2). ¶Determined by counting days from date of earliest illness onset (i.e., event day 1). #Percentage of population targeted to receive prophylaxis who actually obtain and start prophylaxis.

#### Effects of Data Limitations

Because of the underlying model structure, case count inaccuracies are reflected in the final event size projections proportionately to the level of overreporting or underreporting. For example, when the first 3 days of detected cases are 40% underreported in the Sverdlovsk-like scenario (60 cases instead of 100: 12 cases with illness onset event day 1, 6 cases on day 2, and 42 cases on day 3), the median event size caseload was projected to be 698 (range 405–967), 40% less than the 1,164 cases projected in the original scenario.

## Discussion

Our modeling tool provides estimates of future IA caseloads over time and quantifies the effects of various prophylaxis and treatment response options. By integrating projections of the event scale with interventions reflecting healthcare utilization and patient outcomes, our tool permits evaluation of responses during the first days of a real or simulated event.

The accuracy of our FCC projections improves with the number of days of case data available and may provide estimates sufficient for response decisions when 3 days of data are available (Figure 2, panel B). FCC projections made before day 3 probably overestimate the eventual FCC, which may be informative for policymakers (Technical Appendix 2).

The results of our PEP and Healthcare Impact models are consistent with reports showing the benefits of initiating PEP as early as possible after exposure recognition (*3*,*4*,*6*,*7*,*25*). Zaric et al. (*6*) calculated 45.3% event mortality if a 65% effective PEP campaign was completed within 3 days after a 2-day detection delay; our comparable event mortality is 37.1% (by adjusting the associated parameter values in our baseline scenario). In highly effective PEP scenarios, Brookmeyer et al. (*25*) and Baccam et al. (*3*) separately calculated 16%–17% event mortality (we calculated 11% by adjusting our baseline PEP scenario to match theirs), if 100% effective drugs were used after a 2-day delay, 2-day dispensing campaign, 25% final adherence, and 90% inferred uptake (from 90% initial adherence used by Baccam et al., because uptake was not a parameter in either the Baccam or Brookmeyer model).

Unlike prior efforts to evaluate PEP strategies (*3*,*4*,*6*,*7*,*25*), our model includes a PEP uptake parameter in our evaluation of PEP strategies (Table 3; Figure 4). In our model, daily PEP uptake percentage by infected persons deteriorates as the number of unexposed persons requiring PEP increases and when the daily campaign throughput capacity cannot accommodate the increase (because uninfected persons dilute the infected population seeking PEP) (Technical Appendix 2).

The hospital occupancy estimates generated with our Healthcare Impact model are unique among published IA models (Figure 3). This output can support pre-event and intra-event collaboration between public health officials and healthcare system leaders. It also suggests that balancing efforts to allocate countermeasures between public health and healthcare delivery will be a dynamic process that would benefit from daily reassessments of caseloads and responder capabilities.

Our baseline scenario results in lower mortality than was reported for the 2001 US anthrax attacks (37% vs. 45%) (*22*), a result of our assumption of improved treatment effectiveness for persons initiating treatment during the fulminant stage of illness (20% vs. 0) (Table 2). In a large event, in which FCC exceeds treatment resources, treatment effectiveness would deteriorate. Anthrax Assist allows responders to alter effectiveness values (assume crisis standards of care) with regard to local treatment capacity.

Anthrax Assist has limitations. We do not account for gastrointestinal and cutaneous forms of *B. anthracis* infection (Technical Appendix 2). We assume a uniform exposure dosage and a consistent relationship between dose and incubation period across patient types, which may mask logistically relevant temporal variability of illness onset (earlier cases associated with higher inhaled spore counts and vice versa); furthermore, some evidence suggests that certain populations (e.g., children, pregnant women) may be more susceptible to infection or may progress through disease stages differently. Similarly, we do not fully address the consequences of a surge of worried-well patients or the routine demands for healthcare by new and existing patients. Last, although the Centers for Disease Control and Prevention (CDC) Advisory Committee on Immunization Practices recommends anthrax vaccine as part of the PEP regimen (*26*), we do not include vaccine in our PEP Impact model under the assumption that adherence to the full 60-day PEP regimen effectively protects against infection and to assess the effects of decreasing adherence.

Some limitations result from data uncertainties. For example, our Epidemic-Curve model does not pinpoint the timing and location of a release and cannot distinguish between prolonged, short, or multiple releases (Technical Appendix 2). This model is also sensitive to case surveillance uncertainty. To address this uncertainty, Anthrax Assist accepts simultaneous input of up to 3 case series variations. Thus, users can inflate or deflate counts on the basis of perceived underreporting or overreporting, can assign cases to different illness-onset dates, and can examine the influence on outputs. Last, in the absence of a compelling alternative, we rely on the Wilkening analyses of the Sverdlovsk outbreak for our incubation distribution (*13*,*27*), which is not without criticism (Technical Appendix 2). By definition, our Epidemic-Curve model FCC estimates demonstrated high accuracy when applied to the Sverdlovsk-like attack scenario (Figure 2). Use of a Sverdlovsk-like scenario should not be seen as a liability, however, because no evidence suggests that any future IA event would have a substantially different epidemiological profile and our tool permits users to specify other incubation distributions. Because its projections are relatively precise (differences between the highest and lowest FCC estimates are never larger than the estimate itself), Anthrax Assist enables responders to avoid having to consider response options based on event sizes, which differ on a log scale (as with other methods [*8*]).

In conclusion, Anthrax Assist gives public health officials the ability to examine the future scale and consequences of alternative responses to a newly detected anthrax event. This modeling tool mirrors public health practice by using disease surveillance data and permits responders to update projections as new data arrive from the field. The results of our illustrative scenarios underscore the value of integrating epidemic curve projections with decision-based modeling of PEP use and healthcare resource planning. Furthermore, Anthrax Assist highlights the realistic benefit of public health countermeasures and the value of optimizing public perception of PEP.

Technical Appendix 1 The Anthrax Assist modeling tool.

Technical Appendix 2 Additional methods and results associated with the Anthrax Assist modeling tool.
