# Supplementary material for: Modeling Tool for Decision Support during Early Days of an Anthrax Event
Source: Emerg Infect Dis. 2017 Jan;23(1):46–55. doi: 10.3201/eid2301.151787 (PMC5176207; doi:10.3201/eid2301.151787)
Supplement: Technical Appendix 2 — Additional methods and results associated with the Anthrax Assist modeling tool. [file 15-1787-Techapp-s2.pdf]

Article DOI: http://dx.doi.org/10.3201/eid2301.151787

Modeling Tool for Decision Support during Early Anthrax Event

Technical Appendix 2

Further details on the methods used as well as additional results.

Epidemic Curve Model

Calculations: Following Wilkening's 2006 mathematical analysis of the 1979 Sverdlovsk event (*1*), we assume that the incubation periods of inhalation anthrax follow a log-normal distribution in time, with the probability distribution function, $f(t)$, given by:

$$f(t) = \frac{1}{t\sigma\sqrt{2\pi}} e^{-\frac{(\ln t-\mu)^2}{2\sigma^2}}$$

where t is the outbreak event day [1 to 60], and μ is the median incubation period, and σ is the standard deviation. The dose dependence of the incubation period was modeled using the following similar equations for μ and σ:

$$\mu = \alpha + \beta \log(D)$$

$$\sigma = \gamma + \delta \log(D)$$

where D is the exposure dose and $\alpha, \beta, \gamma, and\ \delta$ are parameters with values 10.3, -1.35, 0.804, and -0.079, respectively, derived from Wilkening's least square fit to the Sverdlosk data at the low-end of the dosage spectrum and several nonhuman primate experiments at the high dose-end.

Our methodological approach does not include any mathematical fitting of the incubation distribution to user inputs. Instead, as shown in the equation in the main text, we arrive at the daily and final case counts by combining available case count data (by date symptoms began) with the projected cumulative distribution function (CDF, which is obtained from the original Wilkening Sverdlovsk calculation just above) describing incubation length. For example, if the CDF suggests that by day 3, 20% of cases should have become symptomatic, and 10 total cases

have been detected by day 3, we assume that the observed 10 cases represents 20% of all cases. This results in a final case count of 50 (from 10 x 100 / 20). Our results are discretized, because rather than solving the continuous log-normal function describing incubation distribution length we calculate the proportion of infected that should be symptomatic for each 24-hour time interval on the basis of the selected lognormal incubation distribution. This is in some senses the opposite of the approach taken by Egan et al (*2*), although our methods share fundamental features of with this more sophisticated model (Equation 3.6 in the Egan et al paper). We account for uncertainty (create high/median/low estimates) in this calculation in several ways: 1) we assume various CDFs based on different average inhaled spore counts, and 2) we permit up to 3 case series of data as input. A user can “fit” their case data to these calculations by altering their case series or the average inhaled spore counts so that the projected results align with their entries.

Considerations in choosing exposure values: To illustrate the model, we used a median value of 360 spores/person (range 1–8,000). One spore represents the minimum possible infectious dose and also provides an upper bound on the Final Case Count; 360 spores is the median dosage estimated to have occurred during the 1979 aerosol release of B. anthracis spores in Sverdlovsk, USSR; and 8,000 spores is a plausible high-dose (and therefore lower case-count) estimate (*1*,*3*). For more on the implications of this choice, see the section further below: Further Limitations Detail, Uniform Exposure Dosage.

Considerations in choosing an incubation distribution: The reliance upon the Sverdlovsk event at the low-dose end of the spectrum is seen as one limitation of the 2006 Wilkening incubation model, among the following two others, as cited by Toth *et al.* (*3*) (in a more recent comprehensive review of IA dose response models): Wilkening’s 2006 model is 1) derived mathematically, rather than mechanistically from assumptions having a biological basis (i.e. spore germination and clearance in the lungs), and 2) it assumes an infectivity dose (ID) probability (ID_{50}=8,600) following a report by Glassman (*4*) on data from unpublished work, which does not allow an evaluation of how other model types may fit the data. Toth *et al.* also proposes a model of their own (an exponential mechanistic model with ID_{50}=11,000 [7,200-17,000]) which was shown to be consistent with the Sverdlovsk event’s autopsy-confirmed cases [not the entire set of cases identified by Meselson *et al.’s* (*5*) analysis of the event, upon which the 2006 Wilkening distribution is based]. In 2008, Wilkening published a similar exponential mechanistic model which is indistinguishable from Toth *et al.’s* model, but only when applied to

the first eight days of the Sverdlovsk event (due to Toth's use of just confirmed cases) (*6*). After the eighth day of case reports, it appears that the 2008 Wilkening distribution fits the Sverdlovsk data better. In Wilkening's 2008 analysis, he also compares the fit of his 2006 log-normal model to his 2008 exponential model and claims the following: "a log-normal model fits the data just as well as the [exponential mechanistic model]…Therefore, aside from inferring the value of several parameters associated with disease progression in the host (i.e. lung clearance rate, the spore germination rate, and the bacterial generation time), there would not be much value added". He also goes on to state, that while his exponential mechanistic model was the more accurate model it is also more analytically intractable. Given the apparent equal empiric fit of both model types to the available data and our desire to implement this work in a spreadsheet model using the best, least mathematically intensive method (so that they are more readily understood and accepted by public health officials), we had a preference for Wilkening's 2006 log-normal model.

Influence of capping the event at 60 days: We capped the length of the event at 60 days in order to simplify tool construction and focus graphical output on the event period with most cases. In doing so we prevent 0.02% of infected individuals from contributing to our median projections based on solving the cumulative incubation distribution function at time equals 60 days [result is 99.98]. Capping the event length was irrelevant for our low FCC estimate (all cases occur before then), but in our high FCC estimates (resulting from the lowest calculable exposure assumption of 1 spore) this caused the exclusion of 1.4% of cases (98.6% become symptomatic on or before day 60).

Additional Analyses and Results: To determine the maximal projection accuracy, we also ran the model using the entire 40-day Sverdlovsk-like case series as input. We then compared these results with the actual "Sverdlovsk-like" case count. When the entire Sverdlovsk-like case series is used, the tools projects 701 symptomatic patients (1 more than the actual case count, plausible range: 700-736 cases), mortality of 38% (264 deaths), and a peak hospital caseload of 366 patients on day 19.

Interpreting Accuracy in an Outbreak Situation: Users of Anthrax Assist are cautioned that there may be notable differences between actual case counts and the median estimated cases counts curves. Such differences can be anticipated in the course of model use during an outbreak

(such as when updating the model daily with new case information from the field) as a result of the under-reporting of cases and different sub-populations within the impacted area being exposed to different levels of inhaled spores. A mismatch between the Epidemic Curve projections and the event's line list data may provide a hint that the exposure is not the result of a single point-source one-time release (for which Anthrax Assist is designed). Such issues are not likely to be noted until four or more days of data are used as input, but can be dealt with by altering the values of the average inhaled spore counts, and utilizing different case series as input (Anthrax Assist permits up to 3 case series of data as input) to account for various degrees of potential under-reporting. In such a way a user can "fit" the projections to their case data or their case data to the projections. Such user-controlled adjustments accomplish the type of curve-fitting that can be performed automatically by more sophisticated statistical methods (e.g., the "back-calculation" software of Egan et al [2]).

PEP-Impact Model

Adherence Decay: The proportion of individuals adhering to the prescribed twice daily antibiotic PEP regimen was based on an assumption that adherence degrades linearly between the campaign initiation, where it is 100%, and the user-specified adherence value on the final event day. This structure directly contributed to adherence exhibiting the least influence on averted cases and deaths (shown in the sensitivity analyses results). This was due to most infected already having become symptomatic in the event days before a decrease in adherence (even the most precipitous one) exerted its influence on PEP effectiveness. Even when we lengthened the incubation period to the greatest extent possible (based on an exposure using the minimum possible infectious dose [1 spore]), adherence was still the least influential PEP-related parameter on averted cases/deaths, although the span between the percent of averted cases at 15% and 90% adherence widened from 5% (Table 5) to 8% (the difference between 48% and 56%, respectively for a 1 spore exposure profile). We could not find any evidence to support a different method for modeling the change in adherence over time. One consideration (based on the findings of Egan *et al.* showing a dynamic relationship between the importance of the length of adherence and an event size) was to use the severity and size of the event as positive feedback for adherence (i.e. more deaths = better adherence), but we would be speculating in actually defining such a relationship (*2*).

Additionally, for the sake of simplicity, we assumed that 1) the proportion of individuals not fully compliant with the regimen on their calculated day of becoming symptomatic were 0% protected by PEP, and 2) individuals were 100% protected by PEP if it was taken on their calculated date of becoming symptomatic, even if they stopped taking antibiotics altogether anytime during the next 60 days. The first "simplifying" assumption ignores the potential for partial protection among individuals who have not stopped taking their antibiotics altogether, but who are ingesting less than the prescribed dose over the course of the entire event. During the 2001 Amerithrax event in the US, 42% of postal workers who began taking PEP were classified in this adherence category (*7*). However, estimating the efficacy of a partial dose would have been difficult as we could find no evidence of this in the literature. As such, we felt keeping PEP protection at "all or none" was justifiable. The second "simplifying" assumption disregards the potential for some proportion of the individuals who are initially compliant with the regimen, and who then stop the regimen, to experience a delay in symptomatic illness (i.e. lengthening of the calculated incubation period). This proportion is determined by the inhaled spore count (a higher count requires longer adherence to be protective, as shown by Egan et al. [*2*]) and the shape of our adherence decay curve over time. For example, in an experiment where non-human primates were exposed to ~400,000 spores (1,000 times the median dose in our baseline scenario), Friedlander et al. found that 10% of non-human primates still developed IA after a 30-day doxycycline regimen was completed (*8*). We suspect that in a large inhalation anthrax event (i.e. where the public witnesses illness and death in the community similar to our "Sverdlovsk-like" scenario), that our linear decay in PEP adherence overestimates adherence decay in the initial weeks of the event. Taking Friedlander's results together with our conservative adherence decay rate, we theorize that any diminished impact of PEP resulting from our second "simplifying" assumption is small. Finally, since the influence of each "simplifying" assumption on our projected PEP impact offsets the other, we felt that accounting for the realities they address would unnecessarily complicate the value of the PEP Impact model outputs for decision-makers.

Relationship between the prophylaxis goal and PEP Uptake: In our model, the proportion of infected persons receiving PEP on each day of the campaign decreases as the number of unexposed individuals requiring prophylaxis increases and when the daily campaign throughput capacity cannot accommodate the increase. This occurs because we assume there is no way of

distinguishing infected, asymptomatic individuals from unexposed individuals at the point of dispensation, causing infected individuals to be diluted among the population seeking PEP. As a result, a portion of infected persons will experience a delay in obtaining and starting PEP. In our base case scenario, we assumed public health responders target 500,000 to receive PEP (have enough antibiotics to do so), and can dispense 250,000 regimens daily over 2 days, resulting in 52% of cases averted. For every additional campaign day required to provide PEP to a larger population (using our baseline scenario), responders sacrifice saving 2% to 4% of cases (Technical Appendix Table 1).

Relationship between adherence and PEP Uptake: In our evaluation of the PEP Impact model we compared scenarios with assumptions of both improved uptake and adherence (Scenario 2), or a decrease in both uptake and adherence (Scenario 4), to the baseline PEP scenario (Table 3). It should be noted, however, that in a real IA event, good uptake could be paired with poor adherence and vice versa.

Healthcare-Impact Model

In instances where multiple transition routes out of a single disease/treatment state are possible, our calculations were completed in the following order (each event day): Averted cases were removed from the incubating population each day before determining how many incubating infected transitioned to symptomatic illness; Untreated Prodromals transitioning to fulminant illness were removed before determining the number of prodromals entering treatment; Prodromals in treatment transitioning to fulminant were removed before determining the number recovering; and Fulminants transitioning to death were removed before determining the number of fulminants entering treatment.

Transition rates were selected to approximate the Weibull distribution modeled by Holty *et al.* (see Appendix Tables 3 and 4 in Holty et al) from a systematic review of IA cases since 1900 (*9*). Technical Appendix Table 2 provides a summary of the daily transition rates between all possible disease stages. Our definition of fulminant illness, however, is less severe than the one used by Holty *et al*. (we replace “respiratory failure” with “respiratory distress requiring pleural effusion drainage”), and matches historical definitions: severe symptomatic disease characterized by respiratory distress requiring pleural effusion drainage and/or mechanical ventilation, marked cyanosis, shock, or meningoencephalitis (*10*). This choice does not impact

the overall time in hospital for survivors, which still approximates Holty *et al*.'s distribution, but it may overestimate the length of time patients spend in the fulminant stage of illness. In doing so, our definition of fulminant illness allows for an approach to medical resource planning (based on the census of hospitalized patients in the prodromal and fulminant stages) which errs on overestimating the need for resources to treat advanced IA illness at the expense of underestimating the resources needs for treating early IA illness. As the latter set of resources are likely more abundant or more easily obtained, we felt this the more conservative approach. Users of our model who prefer a different approach, however, may specify a "percent of prodromal patients which recover through fulminant illness" to match the definition of their choice (Table 2).

Public health messaging impact: The timing of public health messaging also impacted CFR, but its influence was limited to an event without a PEP campaign or an ineffective one, due to a logic constraint we imposed on a user's PHM date input: PHM must occur on or before the date of a PEP campaign's initiation because we assume PHM to occur as part of a PEP campaign's "rollout". When the first 3 days of case data were utilized for projections in an unmitigated scenario, and public health messaging was disseminated on the second event day [the base case without a PEP campaign], the Healthcare Impact model projected 51 fewer deaths (a 5% lower CFR) than when messaging occurred 1 week later. CFR improved (decreased) with earlier PHM by improving the ratio of symptomatic individuals seeking treatment in the prodromal illness stage to those seeking treatment in the fulminant illness stage.

Attack Scenario: Sverdlovsk Adaptation:

Our choice of attack scenario stemmed from a desire to illustrate the model with a plausible event that was also large enough to necessitate a wide-scale public health response. As such, we created an attack scenario case series patterned after the 1979 Sverdlovsk (USSR) event and inflated it into a larger event. We created this "Sverdlovsk-like" case series by multiplying each day's case count from the Sverdlovsk event by a factor of 10 (Technical Appendix Figure). Using an historical event, vs. one manufactured mathematically, also avoids the issue of "fractional patients". The resultant scenario was a 40-day, 700-patient case series that matched the daily proportional caseload of the 1979 event.

Further limitations detail

Other forms for anthrax infection: The cutaneous form accounted for half of all identified infections in the 2001 Amerithrax event. Although the cutaneous form is less severe, in a large event it can have healthcare requirement consequences, as up to 20% of cutaneous cases may have similarly intensive treatment requirements as the inhalational form (*11*), and up to 40% could require a hospital bed (based on the percent of cutaneous cases expected to develop "malignant edema", which would require administration of IV steroids and antibiotics) (*12*,*13*).

Uniform exposure dosage: In a real population-wide anthrax event, different populations would likely be exposed to different amounts of aerosolized spores (e.g. based on proximity to a release source or time spent in an exposure zone), and even respond differently to the same exposure amounts, resulting in many different incubation distributions among the populations exposed. We chose to rein in these issues by assuming a singular incubation distribution based on an average exposure dosage (a median value of 360 spores/person [range 1–8,000]), and a consistent relationship between exposure dosage and patient types. These assumptions result in our projections both overestimating and underestimating the rapidity with which some groups of individuals in the impacted population would become symptomatic. Although there is not enough data to quantify the bias introduced from assuming a consistent relationship between dose and incubation across patient type, one could potentially express the direction of the bias on the model's estimate based on any known differences between the demographics in the first cases and the general populace of the impacted region. We chose to use one spore as the minimum possible, average infectious dose, to generate a maximal possible upper bound on the Final Case Count projections We chose 360 spores as the median dosage because it was the dosage estimated to have occurred during the 1979 aerosol release of B. anthracis spores in Sverdlovsk, USSR; and 8,000 spores is a plausible high-dose (and therefore lower case-count) estimate (*1*,*3*). Toth et al. notes that among 13 anthrax modeling papers reviewed, the ID1 (that is, the number of inhaled spores necessary to cause infection in 1% of exposed individuals) ranged from 1 to 9,900 (*3*).Such an exposure profile in an event seems extremely unrealistic (and it can be changed to user's liking), but its use greatly improves the likelihood that this model will overestimate the actual event size. And this is our intention, as it is the authors' opinion, that in a population-wide event, public health practitioners would rather deal with the repercussions of an 'over-response' than the loss of life from being under-prepared.

References

1. Wilkening DA. Sverdlovsk revisited: modeling human inhalation anthrax. Proc Natl Acad Sci U S A. 2006;103:7589–94. http://dx.doi.org/10.1073/pnas.0509551103

2. Egan JR, Hall IM. A review of back-calculation techniques and their potential to inform mitigation strategies with application to non-transmissible acute infectious diseases. J R Soc Interface. 2015;12:•••.http://dx.doi.org/10.1098/rsif.2015.0096

3. Toth DJ, Gundlapalli AV, Schell WA, Bulmahn K, Walton TE, Woods CW, et al. Quantitative models of the dose-response and time course of inhalational anthrax in humans. PLoS Pathog. 2013;9:e1003555. http://dx.doi.org/10.1371/journal.ppat.1003555

4. Glassman HN. Discussion. Bacteriology Review. 1966:657–9.

5. Meselson M, Guillemin J, Hugh-Jones M, Langmuir A, Popova I, Shelokov A, et al. The Sverdlovsk anthrax outbreak of 1979. Science. 1994;266:1202–8. http://dx.doi.org/10.1126/science.7973702

6. Wilkening DA. Modeling the incubation period of inhalational anthrax. Med Decis Making. 2008;28:593–605. http://dx.doi.org/10.1177/0272989X08315245

7. Jefferds MD, Laserson K, Fry AM, Roy S, Hayslett J, Grummer-Strawn L, et al.; Centers for Disease Control and Prevention Anthrax Adherence Team. Adherence to antimicrobial inhalational anthrax prophylaxis among postal workers, Washington, D.C., 2001. Emerg Infect Dis. 2002;8:1138–44. http://dx.doi.org/10.3201/eid0810.020331

8. Friedlander AM, Welkos SL, Pitt ML, Ezzell JW, Worsham PL, Rose KJ, et al. Postexposure prophylaxis against experimental inhalation anthrax. J Infect Dis. 1993;167:1239–43. http://dx.doi.org/10.1093/infdis/167.5.1239

9. Holty JE, Bravata DM, Liu H, Olshen RA, McDonald KM, Owens DK. Systematic review: a century of inhalational anthrax cases from 1900 to 2005. Ann Intern Med. 2006;144:270–80. http://dx.doi.org/10.7326/0003-4819-144-4-200602210-00009

10. Brachman PS. Inhalation anthrax. Ann N Y Acad Sci. 1980;353:83–93. http://dx.doi.org/10.1111/j.1749-6632.1980.tb18910.x

11. Inglesby TV, O’Toole T, Henderson DA, Bartlett JG, Ascher MS, Eitzen E, et al.; Working Group on Civilian Biodefense. Anthrax as a biological weapon, 2002: updated recommendations for management. JAMA. 2002;287:2236–52. http://dx.doi.org/10.1001/jama.287.17.2236

12. Irmak H, Buzgan T, Karahocagil MK, Sakarya N, Akdeniz H, Caksen H, et al. Cutaneous manifestations of anthrax in Eastern Anatolia: a review of 39 cases. Acta Med Okayama. 2003;57:235–40.

13. Kaya A, Tasyaran MA, Erol S, Ozkurt Z, Ozkan B. Anthrax in adults and children: a review of 132 cases in Turkey. Eur J Clin Microbiol Infect Dis. 2002;21:258–61. http://dx.doi.org/10.1007/s10096-002-0704-6

Technical Appendix Table 1. Percent of projected cases averted by PEP associated with increasing the size of the population targeted for prophylaxis (in a jurisdiction of 5 million population)*

Prophylaxis goal	Population targeted to receive PEP (%)	Campaign duration, d	Projected averted cases (% saved)
250,000	5%	1	55
500,000	10%	2	52
750,000	15%	3	48
1,000,000	20%	4	45
1,250,000	25%	5	41
1,500,000	30%	6	38
1,750,000	35%	7	36
2,000,000	40%	8	33
2,250,000	45%	9	31
2,500,000	50%	10	29

*Assumes a maximal 250,000 person daily campaign throughput and that 100% of the exposed population are captured in the targeted population. Estimates were produced using data from the first 3 days of the 1979 Sverdlovsk (USSR) anthrax outbreak (*5*), inflated by a factor of 10. All other values used in calculations, except for the Prophylaxis Goal shown, are provided in Table 2.

Technical Appendix Table 2. Proportions of ill transitioned through Inhalation Anthrax illness stages by the number days in an illness stage and treatment classification (same for individuals who receive and do not receive PEP)

	Untreated		Treated						
			Treatment sought in prodromal stage					Treatment sought in fulminant stage	
Days in preceding illness state	Prodromal to Fulminant	Fulminant to Death	Prodromal to Recovered	Prodromal to Fulminant, who will die	Prodromal to Fulminant, who will recover	Fulminant to Death	Fulminant to Recovered	Fulminant to Death	Fulminant to Recovered
1	0.2	1	0	0.5	0.2	0	0	0	0
2	0.4		0	1	0.4	1	0	1	0
3	0.6		0		0.6		0		0
4	0.8		0		0.8		0		0
5	1		0		1		0		0
6			0.01				0.01		0.01
7			0.03				0.03		0.03
8			0.06				0.06		0.06
9			0.11				0.11		0.11
10			0.19				0.19		0.19
11			0.3				0.3		0.3
12			0.43				0.43		0.43
13			0.56				0.56		0.56
14			0.69				0.69		0.69
15			0.8				0.8		0.8
16			0.88				0.88		0.88
17			0.93				0.93		0.93
18			0.96				0.96		0.96
19			0.98				0.98		0.98
20			0.99				0.99		0.99
21			0.99				0.99		0.99
22			0.99				0.99		0.99
23			1.00				1.00		1.00

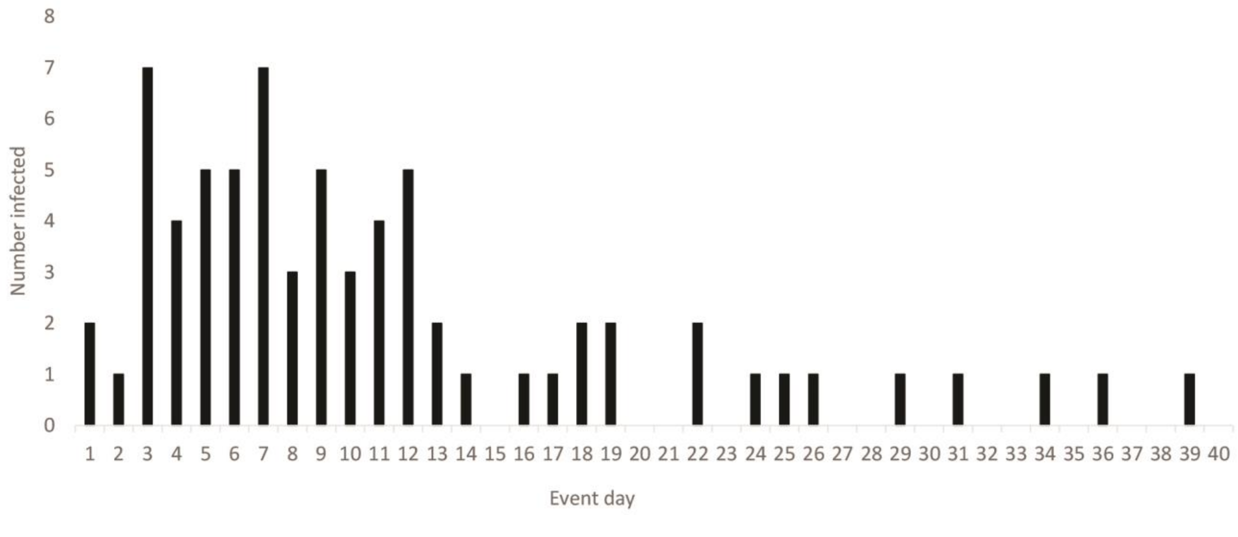

Technical Appendix Figure. Sverdlovsk-like case series for model testing, based on outbreak of inhalational anthrax in Sverdlovsk, USSR, in 1979 (*1*).
